# Insights From Diverse Perspectives on Social Media Messages to Inform Young Adults With Cancer About Clinical Trials: Focus Group Study

**DOI:** 10.2196/64265

**Published:** 2025-01-20

**Authors:** Melissa P Beauchemin, Desiree Walker, Allison Rosen, Maria Frazer, Meital Eisenberger, Rhea K Khurana, Edward Bentlyewski, Victoria Fedorko, Corey H Basch, Grace C Hillyer

**Affiliations:** 1 Columbia University School of Nursing, New York, NY, United States; 2 Afiya, New York, NY, United States; 3 Advocates for Collaborative Education, Santa Cruz, CA, United States; 4 Pediatrics, Hematology/Oncology, University of Iowa Health Care, Iowa City, IA, United States; 5 Cancer Clinical Protocol and Data Management, Columbia University Herbert Irving Comprehensive Cancer Center, New York, NY, United States; 6 Department of Epidemiology, Columbia University Mailman School of Public Health, New York, NY, United States; 7 Department of Public Health, William Paterson University, Wayne, NJ, United States

**Keywords:** young adults, adolescent and young adult cancer, cancer, cancer treatment, clinical trials, clinical trial awareness, clinical trial enrollment, clinical trial knowledge, clinical trial attitudes, clinical trial enrollment barriers, social media, social media messages, psychosocial, United States

## Abstract

**Background:**

Low rates of adolescent and young adult (YA; aged 15-39 y) clinical trial enrollment (CTE), particularly among underserved groups, have resulted in a lack of standardized cancer treatments and follow-up guidelines for this group that may limit improvement in cancer treatments and survival outcomes for YAs.

**Objective:**

To understand and address unique barriers to CTE, we conducted focus groups to learn about informational, financial, and psychosocial needs of YAs surrounding CTE and identify strategies to address these barriers.

**Methods:**

We conducted 5 focus groups in 2023 among a diverse sample of YA patients from across the United States. An interview guide was developed collaboratively with YA advocates. Specifically, informational needs, financial concerns, and psychosocial issues were explored, and participants were probed to suggest strategies, especially those that leverage technology, to address these barriers. Sessions were audio recorded, transcribed, and coded using direct content analysis. Findings were synthesized through consensus discussions.

**Results:**

We confirmed the previously proposed thematic barriers regarding YA CTE and identified 9 subthemes: awareness, lack of clear and accessible CTE information, fear of the unknown, assumptions about costs, insurance coverage, navigating financial responsibilities, clinical trial discussions, clinical trial misconceptions, and desire for a support network. Throughout, YAs mentioned needs that might be addressed through informational outreach leveraging digital technology, the internet, and social media.

**Conclusions:**

This study expands knowledge of YA perceived barriers to CTE. These findings suggest that leveraging digital technology to disseminate reliable information to address needs may be an effective strategy to improve clinical trial participation in the YA population.

## Introduction

Among adolescents and young adults (AYAs; aged 15‐39 y) with cancer [1], clinical trial enrollment (CTE) rates range from 3% to 14%, a number disproportionately below the observed annual cancer incidence in this age group [2]. Previous research shows the disparity in CTE among AYAs is stark when compared to the cancer incidence rates across other age groups. For instance, pediatric and older adult patients demonstrate higher CTE rates compared to AYAs, although AYAs experience higher cancer incidence [3-5]. Despite cancer incidence being 3 times greater and with lower survival rates across all major tumor types for AYAs [3-8], there remains insufficient progress in discovering and developing new therapies, investigating molecular differences, and CTE for this population [9,10]. Inferior survival outcomes are further compounded among AYAs from marginalized groups (eg, non-White, publicly insured, low-income, rural residents, and non-English speakers [9,11]).

Young adults (YAs; aged 18-39 y) with cancer are a distinct group with needs that differ greatly from pediatric and adult populations. A cancer diagnosis as a YA can derail developmental transitions and impact emotional, cognitive, and social development [12,13]. As digital natives, a term used to describe individuals who have grown up with digital technology as an integral part of their daily lives, YAs approach health information-seeking differently than their younger and older counterparts [14]. Their proficiency with digital technology makes them especially adept at accessing medical information online using a wider variety of digital resources than older adults [15,16]. Interventions to increase CTE have demonstrated feasibility and acceptability among YAs with cancer [17], however, interventions developed thus far incorporate little input from YA patients.

Abrahão et al [18] explored CTE barriers from the perspectives of physicians and AYA patients using individual interviews and found that poor understanding of clinical trials, financial issues, and psychosocial problems were the greatest obstacles to CTE for AYAs. As the first phase of a larger study (TALK TO ME: Tailored YA Trial Messaging, HF CU22-2020), we conducted focus groups (FGs) to capitalize on interpersonal communication among AYAs with cancer and inform the development of tailored, relevant, and salient social media messages to address barriers to CTE among YAs with cancer.

## Methods

### Study Design

To build on prior work understanding barriers and facilitators to CTE among YAs, we used a qualitative descriptive approach to conduct FGs among YAs with cancer, as this methodology is ideal for providing a rich description of an experience by participants rather than interpreting or theorizing [19,20]. As the goal of this phase of this study was to develop social media messages for YAs regarding CTE, this methodologic approach was ideal to stay close to the participant experiences and perceptions. We used FGs as the mode of data collection to tap into the communication, interactions, and processes that are more readily observed among groups than in individual interviews [21].

### Study Population and Recruitment

FG participants were recruited by leveraging the networks and social media platforms (eg, Facebook support groups and Instagram) of this study team’s YA advocates via social media posts or by direct outreach. Outreach messages explained that we were recruiting individuals who were diagnosed with cancer between 18 and 39 years of age to participate in a FG to explore YA perceptions about clinical trials with the goal of informing the development of social media messages that aim to improve understanding and address concerns about clinical trials for YAs with cancer. Interested respondents were asked to provide basic sociodemographic (current age, gender identity, race or ethnicity, and city or state of residence, rural vs urban), whether they were active social media users, clinical information, including age at diagnosis and cancer type and availability (weekday vs weekend and daytime vs evening) for group participation, via a Google Forms survey link. As we sought to understand reasons for both participation and nonparticipation in cancer clinical trials, participation in a clinical trial was not an eligibility criterion.

This study team’s YA advocates contacted potential participants to explain this study’s procedures, confirm eligibility (diagnosed with cancer between 18 and 39 years of age, active social media user, and English speaker), and invite participation. Potential participants were then purposively sampled to represent a range of experiences (1 group all female and any race or ethnicity; 1 group all male and any race or ethnicity; 3 groups mixed gender identity, race or ethnicity, and any cancer type).

### FG Data Collection

An FG guide was developed during weekly study team meetings guided by the Abrahaō framework from individual interviews among AYA participants [18]. We applied an iterative process to the guide development; pilot testing was carried out by this study’s team members, including patient advocate partners [22]. To ensure study participants were oriented to the topic, a brief, 2‐ to 3-minute overview was prepared by this study team to introduce and define a clinical trial [23].

FGs were conducted virtually over Zoom between May and October 2023 and were 1 to 1½ hours in duration. Moderators included female YA advocates and, for the all-male group, a male oncology nurse practitioner. An information sheet was distributed via email to all participants before the FG.

The sessions began with a brief explanation of clinical trials and important definitions. Participants shared their experiences with cancer clinical trials including experiences of family members or friends and were asked about reasons for participating or not, and the support needed for trial involvement specifically as related to information, finances, and their psychosocial needs [18]. They also discussed their trust in social media information, preferred platforms, where they often seek health information, and suggested ways to use it to share clinical trial information with YAs.

### Data Analysis

All FG sessions were audio-recorded and transcribed verbatim using NVivo Transcription, Lumivero, LLC. Participants were assigned a speaker ID, and transcripts were deidentified and cleaned using a denaturalized approach [24] to facilitate analysis of the participants’ words rather than a more interpretive approach. We used a directed content analysis [25] to enrich and expand our understanding of barriers and facilitators to refine existing themes rather than identify new ones. Study team members trained (MPB, ME, RKK, and GCH) in qualitative analysis coded the transcripts first individually; then consensus discussions were held to discuss coding discrepancies, synthesis of findings, and to ensure intercoder agreement. Saturation was anticipated given the deductive nature of the analytic approach but was tracked during consensus discussions [26].

### Ethical Considerations

Verbal consent, including consent to be audio recorded, was obtained at the start of each FG. Participants were informed of their right to withdraw from this study at any time without penalty. For their time and effort, participants were compensated with a gift card valued at US $50. Regulatory approval was obtained from Columbia University Institutional Review Board (ID# AAAU4337).

## Results

### Overview

A total of 33 individuals participated in the FGs (maximum 8, minimum 4). The mean age was 32.2 (SD 5.7) years. Males and females were equally represented; 42.4% (n=14) were White and 42.4% (n=14) were Black or African American. Most (n=24, 72.7%) resided or sought treatment in urban areas, and 27.3% (n=9) were diagnosed with breast cancer (Table 1). Findings are grouped by themes (informational, financial, and psychosocial) and subtheme. Exemplary quotes further supporting subthemes are displayed in Table 2.

**Table 1.** Characteristics of focus group participants (N=33).

Demographic	Value
**Age (years)**	
Mean (SD)	32.2 (5.7)
Range	24-42
**Gender, n (%)**	
Female	17 (51.5)
Male	16 (48.5)
**Race, n (%)**	
White	14 (42.4)
Black or African American	14 (42.4)
Asian	1 (3)
Mixed race	3 (9.1)
Did not specify	1 (3)
**Rural versus urban, n (%)**	
Rural	9 (27.3)
Urban	24 (72.7)
**Cancer type, n (%)**	
Skin cancer	2 (6.1)
Breast cancer	9 (27.3)
Lymphoma	2 (6.1)
Other	20 (60.6)

**Table 2.** Interview themes and categories with additional illustrative quotes.

Theme and categories		Representative quote
**Theme 1: Information or knowledge**		
	Awareness	“I was given two options as far as radiation and chemo. And basically, they were like, these are the two options you have, which one do you want? Clinical trials never even brought up.” [FG1^a^]
	Lack of standardized or central system	“One of the biggest issues, I think upon diagnosis as a patient and as a as a caregiver is information overload.” [FG1]
	Fear of the unknown	“Being reminded that...my health is more important than just a number or an experiment or a statistic. And then in terms of. Like in a form of social media that I would go to, I think like visual media tends to be more compelling to me like to be able to see your face and and a name and to know you know who that person is giving me information.” [FG4]
**Theme 2: Psychosocial**		
	Clinical trial discussions	“You know, being upfront about the risks obviously is important, but the potential benefits too, I think just prevent presenting balanced information about the nature of the trial is probably all you can do, really.” [FG5]
	Clinical trial misconceptions	“I feel like clinical trials have been sort of this last-ditch effort after thought” [FG1]
	Desire for support network	“Social media is the connection to community” [FG2]
**Theme 3: Financial**		
	Assumptions or knowledge about clinical trials	“And another one said, In order to find out if you’re eligible, you need to pay $750 out of pocket to do this consultation. So that just makes people I mean, it just it’s just not fair.” [FG1]
	Young adult specific concerns	“The trial sponsor may be able to offer some form of compensation or some sort of resource, but there’s also the sort of unintended financial impact that can happen, especially to this age group taking time off from work, finding childcare, meeting the needs for other bills, that kind of stuff.” [FG1]
	Communication	“And I think that the visits they go by so quickly anyways, there’s so much to cover. There probably wouldn’t even be enough time for a provider to sit down and have a full conversation about what is a clinical trial.” [FG5]

a FG: focus group.

### Theme 1: Informational

#### Subtheme 1: Awareness

Across all FGs, YAs cited varying levels of exposure to information and lack of awareness of cancer generally, their specific cancer, and treatment options that may have included clinical trials. Many reported facing the challenges of gathering important information about their diagnosis and treatment options on their own and conveying their informational needs to others. One participant reflected saying, “when I was diagnosed, I was still on my parent’s health insurance and my dad handled all of the insurance...I didn’t know anything like, I feel like if I hadn’t had that support, like I can’t even imagine.”

Participants also noted that they wanted to understand their disease and treatment options to better participate in their own care, however finding relatable and accessible information was difficult. One participant remarked “I thought I was really educated until I got diagnosed with cancer and then I just felt like an idiot.” Some participants felt that they were tasked with processing information about their cancer diagnosis and treatment that was not easily digestible or so complex that “no normal person is just going to know.”

The process of deciding on treatment, whether the standard of care or a potential clinical trial, was described as “draining.” In some cases, participants said that they were completely unaware that a clinical trial may have been an option. As one person recalled, “I was given two options as far as radiation and chemo. And basically, they were like, these are the two options you have, which one do you want? Clinical trials [were] never even brought up.”

More specifically, YAs wanted to be better informed about their possible treatment options and how they might impact them in the future. They expressed the need to comprehend the risks and benefits associated with each treatment option to make an informed decision. One participant summarized this as “there’s so many different things that you have to consider when making these decisions and side effects are no joke for cancer treatment…there’s just it’s stuff that’s very lasting and those are other things you have to consider when making the decision.” Most importantly, participants conveyed that they wanted their providers to consider them as a whole person when treating their cancer, specifically considering their reproductive health concerns and fertility preservation. One participant told us “I had like seven days to decide if I wanted to freeze my eggs or not.” They believed that the provider should make these YA-unique issues a top priority when creating cancer treatment plans and ensure that YAs fully understand these implications at the start.

#### Subtheme 2: Lack of Standardized or Central System

Participants repeatedly spoke of the difficulty in finding centralized databases with information on their cancer or resources relevant to their experiences. When trying to understand the concept of a clinical trial, participants found the information to be complex and filled with medical terminology they did not understand only added to the stress of their illness. Many felt intimidated, overwhelmed, and frightened by the information they found, especially for YAs with rare cancers. Among participants who searched these databases, the consensus was there needs to be “some sort of database that like pulls that information [together]...clinical trial wise…it would be nice to know that there are options for people that have like, not so ideal cancer circumstances.” Additionally, participants felt this information should be engaging, compassionate, and written in plain language.

When researching clinical trials, YAs said that they would like information to come from trusted sources such as their medical team or the organization implementing the clinical trial. It helps, they said when “someone is maybe [a] medical personnel…or a governmental organization[s] that you can then be able to trust that information.” One participant who participated in a clinical trial explained that “the biggest differentiator for me was my oncologist explaining to me all of the options for clinical…and really went into detail…he explained to me in a way that I could understand...and I trusted him. I trusted him implicitly.”

Participants addressed the role of caregivers seeking treatment information for their YAs. They emphasized the need for resources to comfort both YAs and their caregivers throughout treatment.

The caregiver is often the person doing this research or supporting this. And so, I think it’s really important to consider them as your equal audience.

They also warned that “One of the biggest issues, I think upon diagnosis as a patient and as a caregiver is information overload.”

#### Subtheme 3: Fear of the Unknown

A common fear cited by participants regarding clinical trials was adverse side effects of the experimental treatment. They also wanted to connect with others like themselves to hear firsthand about their experience with cancer and the treatment options they were considering to put their minds at ease. They stated that when they could not access the support and information they needed through their care team, they sought it on social media using Instagram hashtags and Facebook groups.

I think the biggest thing for me, where I found the most helpful information was searching hashtags on Instagram. I searched my chemo regimen hashtags. I searched my diagnosis. I searched by stage. I searched AYA cancer...And I would find people there that would have a community on their pages and resources on their pages.

YAs that used social media to supplement their understanding and find support expressed concern over the lack of reliable sources and the spread of misinformation on some platforms. Participants suggested that it is difficult to differentiate between helpful information and potentially harmful content on social media. Another issue related to the use of social media reported by participants was “doom scrolling,” which can cause increased conflict within the patient. One participant explained that “because of the information overload, I’ve really had to limit my information intake from social media.”

### Theme 2: Psychosocial Issues

#### Subtheme 1: Clinical Trial Discussions

YAs cited that care teams should remember that patients usually have limited knowledge of clinical trials, including the risks and benefits. As one participant explained, “I actually refused [experimental] treatment because I thought my only option was chemotherapy and radiation...if I knew that I had other options, I probably would have been more proactive, no matter what the cost was like.” Participants stated that initiating simple dialogue that normalizes CTE in the context of cancer treatment would motivate patients to consider clinical trials as a treatment option.

#### Subtheme 2: Clinical Trial Misconceptions

Participants explained that common misconceptions about clinical trials were that they were reserved for patients with advanced disease and considered a “last ditch effort” for only those with terminal disease. These misconceptions add to the fear surrounding clinical trials and can present as a key barrier to CTE and psychological distress. Participants explained that providers should anticipate and address misconceptions about clinical trials during treatment discussions.

#### Subtheme 3: Desire for a Support Network

Participants reported that the effects of their illness and treatment took a significant toll on their mental health and that additional check-ins with the medical team would have been beneficial. For those who participated in a clinical trial, the additional contact and attention by the clinical trial care team eased the burden. One participant who participated in a clinical trial explained that “the additional support for me was one of the best things that came out of the clinical trial because I was at such a large hospital…This connection is especially important during trial enrollment as this is an uncertain time for patient[s].”

Participants explained that they would have appreciated a peer support network while navigating life and treatment simultaneously. As one participant explained, “cancer is the worst part-time job on the entire planet.” They use social media as a “connection to community.” Participants expressed interest in a social media account or online group that connects patients more easily since it can be valuable for communication with those who have had similar experiences.

Some participants mentioned that discomfort while considering a clinical trial can be further exacerbated by family members’ lack of understanding or distrust of medical research. This may lead family members to caution patients away from participating. One family responded to the idea of a clinical trial their son was considering by saying “No, don’t do it. You know, they’re going to do x y z. It’s not safe. You know, you’re not that sick.” The participant explained that their family saw a clinical trial as “a last-line option and not a first-line option.” The importance of providing caregivers and others surrounding the patient with accurate information to support the YAs treatment decisions was stressed.

### Theme 3: Financial Concerns

#### Subtheme 1: Assumptions About Clinical Trials Costs

Financial hardship was mentioned by many participants as a key deterrent to CTE. Several reported that the fear of financial problems prevented them from inquiring about clinical trials because they assumed resources would not be provided or that the resources provided would not be sufficient to cover additional costs. Patients applauded treatment centers that provide financial navigation and resources to patients considering clinical trials. They believed this could ease barriers such as travel expenses and limited insurance coverage and potentially increase CTE.

#### Subtheme 2: Insurance Coverage

Some participants indicated that the insurance framework is difficult to navigate on its own and can be further complicated by seeking nonstandard of care treatment. One participant explained that they “wanted to get enrolled in a clinical trial that was at another health institution” but they did not proceed because “my insurance wouldn’t cover it because it was out of network.” Alongside this, the struggle to balance their schooling, family, and work responsibilities impacts the state of their finances.

#### Subtheme 3: Navigating Financial Responsibilities

Some of the participants assumed that the patient was responsible for additional costs which deterred them from inquiring about clinical trials as a treatment option. Many participants were unaware that most health insurers cover many, if not all, costs associated with clinical trials, that costs related to increasingly more clinical trials are covered by the sponsor, or that there are occasions when sponsors offer financial assistance to address costs not covered by insurance or the clinical trial. One participant remarked, “I think a lot of us don’t even realize that our hospital might have financial navigators or patient navigators who exist to help answer those questions.” YAs believed providers and any online sources should educate patients about their financial options while addressing common misperceptions.

## Discussion

In this study, we explored categorical individual-level barriers [18] to CTE among a diverse group of individuals diagnosed with cancer as YAs to extend our knowledge and inform future strategies to address these barriers. Through FGs representative of gender identity, race, and geographic region in the United States, we found that lack of knowledge and imperfect communication were the greatest barriers to CTE in this population. Participants indicated that limited awareness about clinical trials, commonly held misperceptions, misinformation on websites and social media, and a lack of clear and accessible information about clinical trial risks and benefits likely contribute to underrepresentation among YAs. Proactively providing anticipatory guidance may circumvent distress if the topic is raised and reduce patient hesitancy in participation.

Consistent with other literature, our study supports that YAs often seek information on the web or social media [27,28] and participants favored an intervention using social media to address CTE barriers. They also provided clear guidance on how information might be tailored to YAs and what specific information may be most helpful in addressing concerns and barriers to CTE.

In many ways, the financial concerns and psychosocial issues reflected a subset of the informational gaps reported by participants. Finances were a significant concern, and several participants were unaware of insurance coverage requirements or that resources such as financial navigators exist to help with the costs of CTE [29]. Having financial information readily available during the cancer treatment decision-making process can support discussions around a clinical trial option. A digital support network may also provide a connection with others sharing a similar lived experience during times of loneliness and fear.

We followed rigorous qualitative methodology to conduct our study, however, there are limitations to acknowledge. Potential selection bias may be present, as participants were recruited through our YA patient advocates on the internet and, thus, may have been more technologically proficient and familiar with social media and clinical trials than the general YA patients with cancer community. However, the vast majority of YAs have an online presence and use social media and the internet regularly. Further, as with other qualitative research, the transferability (ie, generalizability) of these data may be limited although great effort was expended to ensure that our sample was diverse in terms of sociodemographic characteristics to yield FGs representative of the YA population. More than half of our FG participants were non-White which provides a deeper view of experiences from underrepresented YAs regarding engagement with and perceptions of CTE. Although we based our discussions a priori on themes identified by Abrahão et al [18], moderators followed the discussions and probed further when any topic arose organically to ensure all potential barriers were explored and thus, resulted in the expansion of subthemes described.

Our research enhances our understanding of the obstacles that YAs face when making decisions about cancer treatment, including the option to participate in a clinical trial. It also provides further evidence that using a combination of approaches at different levels may be most effective in engaging YAs in discussions about CTE. While barriers to CTE at the provider, institutional, and system levels have been extensively studied, the YAs in our study highlight individual-level challenges that need attention. Our findings indicate that a social media intervention focused on sharing reliable information about CTE could help YAs process complex information and make informed treatment decisions, thus improving CTE in this population. Engaging with YAs through social media allows researchers and medical professionals to connect with them in a way that is familiar and comfortable. Further research should address the gaps in information identified in our study to develop resources and educational materials that can enhance understanding of CTE among YAs, their families, and caregivers. When developing treatment plans, it is crucial to prioritize patients’ informational needs and provide all YAs with the tools and resources to have meaningful discussions with their health care providers about CTE.

## Abbreviations

AYA: adolescent and young adult
CTE: clinical trial enrollment
FG: focus group
YA: young adult
